# Depth Reconstruction from Single Images Using a Convolutional Neural Network and a Condition Random Field Model

**DOI:** 10.3390/s18051318

**Published:** 2018-04-24

**Authors:** Dan Liu, Xuejun Liu, Yiguang Wu

**Affiliations:** 1 Faculty of Geomatics, East China University of Technology, Nanchang 330013, China; 2 Key Laboratory of Virtual Geographic Environment, Nanjing Normal University, Nanjing 210023, China; yiguang.wu@hotmail.com

**Keywords:** depth reconstruction, single image, convolutional neural network, conditional random field

## Abstract

This paper presents an effective approach for depth reconstruction from a single image through the incorporation of semantic information and local details from the image. A unified framework for depth acquisition is constructed by joining a deep Convolutional Neural Network (CNN) and a continuous pairwise Conditional Random Field (CRF) model. Semantic information and relative depth trends of local regions inside the image are integrated into the framework. A deep CNN network is firstly used to automatically learn a hierarchical feature representation of the image. To get more local details in the image, the relative depth trends of local regions are incorporated into the network. Combined with semantic information of the image, a continuous pairwise CRF is then established and is used as the loss function of the unified model. Experiments on real scenes demonstrate that the proposed approach is effective and that the approach obtains satisfactory results.

## 1. Introduction

Measuring the depth of a scene is a significant topic of research in photogrammetry and computer vision, which plays an essential role in various applications in 3D reconstruction, video surveillance, and robotics, etc. Much prior work has performed depth acquisition from multiple images taken in accordance with certain requirements [1,2,3], but in fact, many photos may well be not taken by photogrammetric purposes, but rather taken by the public or amateur photographers. Scene structure cannot be correctly recovered through the traditional photogrammetry, due to lack of corresponding features, or too big or small a baseline between these images. Moreover, there usually exists only a single image of a scene, such as historic photos and images from the Internet. Therefore, depth reconstruction from a single image is a basic task with important research value in photogrammetry and computer vision.

The task is an ill-posed and inherently ambiguous problem, as one given image may correspond to an infinite number of possible real world scenes [4]. Therefore, depth acquisition from a single image it is a still challenging issue. Some previous works solve this problem using some depth cues such as geometric characteristics [5,6,7,8], shading [9], texture [10] and contour [11]. However, these works only infere the relative depth of the scene from an image but can’t get the absolute depth. In recent years, many researchers have applied machine learning to the problem and obtained some good results [12,13,14,15,16,17]. A common characteristic of these methods is that they rely on hand-crafted features. Saxena et al. [12] extracted three local features from images: haze, texture variations and texture gradient. Shape- and location-based features were added in [13] for better feature representation. However, these low-level features are still not enough to predict the exact depth values of pixels in an image. Based on Saxena et al. [13], Liu et al. [14] used semantic labels to guide depth reconstruction from a single image. Another challenging problem of these methods is how to utilize extracted image features to measure the depth of each pixel in the image. Many of these methods use a Markov Random Field (MRF) to build the relationships between image features and depth. Unfortunately, it is sensitive to multicolored objects in the image, and involves many assumptions to make the decision.

Recently, the Convolutional Neural Network (CNN) method has become a mainstream of image processing research. Compared with those traditional methods applied to depth reconstruction, CNN can learn a high-level of representation automatically without any manual interventions. Eigen et al. [18] used a multi-scale deep network to estimate depth maps from a single image. To perform pixel-level depth inference, Hu et al. [19] trained a CNN with raw RGB image patches cropped by a large window centered at each pixel. Li et al. [20] presented a framework for depth estimation from a single image, which consists of depth regression on superpixels via a deep CNN model and refining from superpixels to pixels via a hierarchical Conditional Random Field (CRF). Similarly, Wang et al. [21] performed depth prediction via regression on CNN model, combined with a post-processing refining step with a hierarchical CRF, but they joined depth and semantic inference, considering that the two problems are mutually beneficial. Unlike the above methods, Liu et al. [22,23], Xu et al. [24] formulated depth prediction as a continuous CRF learning problem, and used a CNN model to learn the feature representation of the image. The approach combined the strength of the CNN and CRF in a unified framework. However, they ignored the importance of semantic information to depth reconstruction and did not resolve depth ambiguities of a scene.

In this paper, a unified CNN framework is presented for depth reconstruction from a single image, joining a CNN and a continuous pairwise CRF model. A deep CNN network is firstly designed to automatically learn a hierarchical feature representation of the image. To get local details of the image, relative depth trends of local regions inside the image are integrated into the CNN network. Then, a continuous pairwise CRF is established as the loss function of the unified model through semantic information of a scene and the results of the CNN network in the first step. Depth reconstruction is formulated as a CRF learning problem and can be solved by maximum a posteriori (MAP) inference.

## 2. Methods

**Figure 1:**
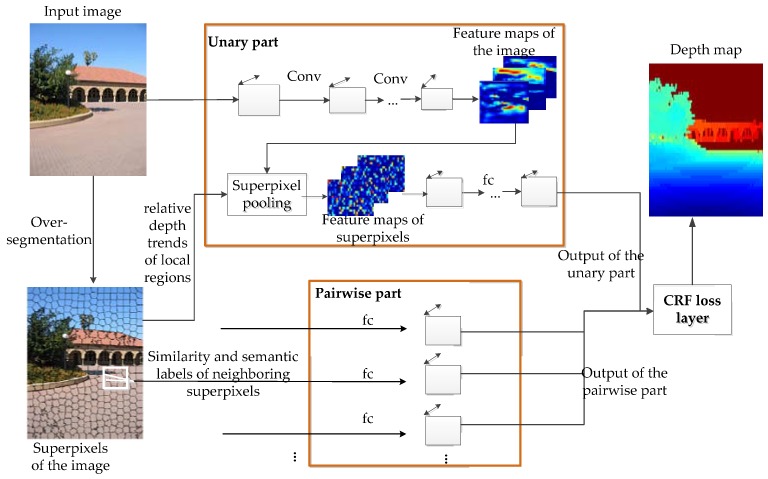
The overall framework of the unified CNN model.

The approach performs pixel-level depth reconstruction from a single image in a unified CNN model framework, shown in Figure 1. The unified model joins a CNN and a continuous pairwise CRF, in which the continuous pairwise CRF is used as the loss function of the CNN. The model architecture consists of three parts: a unitary part, a pairwise part and a CRF loss layer. (1) In the unitary part, a convolutional network is used to obtain convolutional feature maps from the input image. To get feature maps of the superpixels, the convolutional feature maps are fed into a superpixel pooling layer along with the superpixels inside the image. These feature maps are then followed by three fully-connected layers. (2) In the pairwise part, sematic information and similarities of neighboring superpixels are considered and are fed into one fully-connected layer to produce the output. (3) In the loss layer, a continuous pairwise CRF is used as the loss function of the unified CNN framework, which is established via the outputs of the unary and pairwise part. 

### 2.1. Depth Reconstruction Using CRF Model

Given an image $x=\left\{x_{1},x_{2},\cdots ,x_{n}\right\}$ with corresponding to depth labels $y=\left\{y_{1},y_{2},\cdots ,y_{n}\right\}$, where *n* indexes superpixels via over-segmentation, the pairwise CRF are modeled as:

(1) $$P\left(y\;|\;x;\vartheta \right)=\frac{1}{Z\left(x,\vartheta \right)}exp\left\{-E\left(y,x;\vartheta \right)\right\}$$

where ϑ are model parameters and $Z\left(x,\vartheta \right)=\int_{y}exp\left\{-E\left(y,x;\vartheta \right)\right\}\;dy$ the normalization term. The energy E(y,x;ϑ) over superpixels N and edges S takes the following form:

(2) $$E\left(y,x;\vartheta \right)=\sum_{p\in N}\varphi \;\left(y_{p},x;\vartheta \right)+\sum_{\left(p,q\right)\in S}\phi \;\left(y_{p},y_{q},x;\vartheta \right)$$

where $\varphi \;\left(y_{p},x;\vartheta \right)$ and $\phi \;\left(y_{p},y_{q},x;\vartheta \right)$ represent the unary and pairwise potentials respectively.

Once the parameters ϑ are learned, depth map of an image can be predicted by MAP inference, written as:

(3) $$y^{*}=\underset{y}{\arg\max}P\left(y\;|\;x;\vartheta \right)$$

### 2.2. Unitary Part

The unitary part to obtain depth regression of each superpixel in the image uses a deep CNN model for learning feature representation of all the superpixels. The unitary potential $\varphi \;\left(y_{p},x;\vartheta \right)$ of the CNN model is defined as a Euclidean loss associated with the ground-truth depth value $y_{p},p=1,2,\cdots ,n$ and the predication $z_{p}$:

(4) $$\varphi \;\left(y_{p},x;\theta \right)={\left(y_{p}-z_{p}\left(\theta \right)\right)}^{2}$$

**Figure 2:**
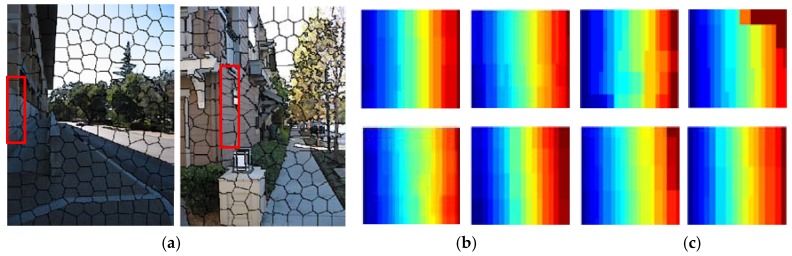
Some local regions of similar relative depth trends from a same sematic label. (**a**,**b**) Some different local regions (superpixels) from a same sematic label (in the red box); (**c**) relative depth trends of the local regions in (**a**,**b**) are similar.

Usually, the depth of a superpixel is calculated with a single value. However, it is too coarse since depth values of different pixels inside the superpixel may be different. Fortunately, there are many local regions with similar structure from a sematic class, which means that their relative depth trends are nearly same, shown in Figure 2. Therefore, the relative depth trends from the same semantic class can be expressed with a limited normalized depth map called a depth template. A normalized depth map of a superpixel is calculated by the depth value of superpixel centers and scale factors. Given the normalized depth map $t_{p}$, the depth value at the superpixel center $c_{p}$ and the scale factor $s_{p}$, the depth map of the superpixel can be defined as: $z_{p}=s_{p}t_{p}+c_{p}$.

To obtain depth templates for each semantic label, the normalized depth maps of all the superpixels with the same sematic label are clustered. In this paper, relative depth trends of the superpixels, which are represented by the depth templates, are incorporated into the CNN network. To obtain the absolute depth values of each pixel inside a superpixel, the outputs of the CNN network for the unary part are designed as the depth value at the superpixel center and its normalized scale factor. The structure of the CNN model is similar to that described by Liu et al. [23], but their outputs are different because this paper joins the relative depth trends of the superpixels.

### 2.3. Pairwise Part

The pairwise part considers the depth relationships between neighboring superpixels, combined with their similarity and semantic information. The pairwise potential of the CRF model is constructed as:

(5) $$\phi \;\left(y_{p},y_{q},x;\beta ,w\right)=\frac{1}{2}R_{pq}{\left(y_{p}-y_{q}\right)}^{2}+\frac{1}{2}w\;\left(l_{p},l_{q}\right)\;{\left(y_{p}-y_{q}\right)}^{2}$$

where, β,w are parameters. The first term $\frac{1}{2}R_{pq}{\left(y_{p}-y_{q}\right)}^{2}$ represents the consistency information of the neighboring superpixels p,q with their similarity matrix $S_{pq}^{}$. $S_{pq}^{}$ is established with color in LUV space, color histogram and texture of Local Binary Pattern. $R_{pq}$ is produced by one fully-connected layer with $S_{pq}^{}$, defined as:

(6) $$R_{pq}=\beta^{{}^{T}}\left[S_{pq}^{\left(1\right)},S_{pq}^{\left(2\right)},\cdots ,S_{pq}^{\left(K\right)}\right]=\sum_{k=1}^{K}\beta^{k}S_{pq}^{\left(k\right)}\;(K=3)$$

The second term $\frac{1}{2}w\left(l_{p},l_{q}\right)\;{\left(y_{p}-y_{q}\right)}^{2}$ in Equation (6) represents the depth smoothness of the neighboring superpixels p,q with their semantic labels. Here $l_{p},l_{q}$ are respectively the sematic labels of p,q and $w\left(l_{p},l_{q}\right)$ represents the semantic weight between them. The higher the weight value is, the smoother the depth between the neighboring superpixels is. A weight matrix w is formed with all the sematic weights. w is a C×C matrix, where C is the number of the sematic labels in the scene. In the weight matrix, $w\left(l_{p},l_{q}\right)$$\left(l_{p}=1,\ldots ,C;l_{q}=1,\ldots ,C\right)$ represents the semantic weight of the semantic labels $l_{p},l_{q}$, and $w\left(l_{p},l_{q}\right)=w\left(l_{q},l_{p}\right)$.

### 2.4. CRF Loss Layer

The loss function of the depth reconstruction model uses the negative log-loss of the pairwise CRF, shown in Equation (1). According to Equations (4) and (5), the potential of the CRF can be expressed as:

(7) $$E\left(y,x;\vartheta \right)=\sum_{p\in N}{\left(y_{p}-z_{p}\left(\theta \right)\right)}^{2}\;+\frac{1}{2}\sum_{\left(p,q\right)\in S}R_{pq}\;{\left(y_{p}-y_{q}\right)}^{2}+\frac{1}{2}\sum_{\left(p,q\right)\in S}w\left(l_{p},l_{q}\right)\;{\left(y_{p}-y_{q}\right)}^{2}$$

Then Equation (1) can be written as:

(8) $$Loss=-\log P\left(y\;|x\;;\vartheta \right)=-\log\left(\frac{1}{Z\left(x,\vartheta \right)}exp\left\{-E\left(y,x;\vartheta \right)\right\}\right)$$

Here ϑ={θ,β,w} are parameters that can be learned by minimizing Equation (8).

## 3. Results

The proposed method is evaluated on the Make3D dataset [12]. The Make3D dataset contains 534 images of outdoor scenes composed of eight semantic classes including sky, tree, road, water, grass, building, mountain and foreground objects. The method is quantitatively evaluated by several common measures used in prior work [20,23]:

(1) mean relative error (Rel): $\frac{1}{T}\sum_{i\in T}\frac{\left|d_{i}-d_{i}^{*}\right|}{d_{i}}$
(2) root mean squared error (Rmse): $\sqrt{\frac{1}{T}\sum_{i\in T}{\left(d_{i}-d_{i}^{*}\right)}^{2}}$
(3) mean log10 error (Log10): $\frac{1}{T}\sum_{i\in T}\left|\log10\left(d_{i}\right)-\log10\left(d_{i}^{*}\right)\right|$

where $d_{i}^{*}$ is predicted depth at pixel i, *d_i_* is the corresponding ground-truth depth, and T is the number of pixels in the image.

As pointed out in [17], the range of pixels in Make3D is limited to a depth range of 0~81 m, due to the limited range and the resolution of the sensor. As done in [17], two criteria are used to measure the errors: (1) C1 errors are calculated with pixels of the ground-truth depth less than 70 m; (2) C2 errors are computed with all pixels in the image.

To evaluate the quantitative results of the proposed method, several state-of-the-art methods are used for comparison. Additionally, considering the influence of the constraint information including sematic information, relative depth trends and CRF on the results, experiments with the dataset are performed, which share the same model with the proposed approach except integrating the constraint information.

### 3.1. The Experiments with Different Constraint Information

**Figure 3:**
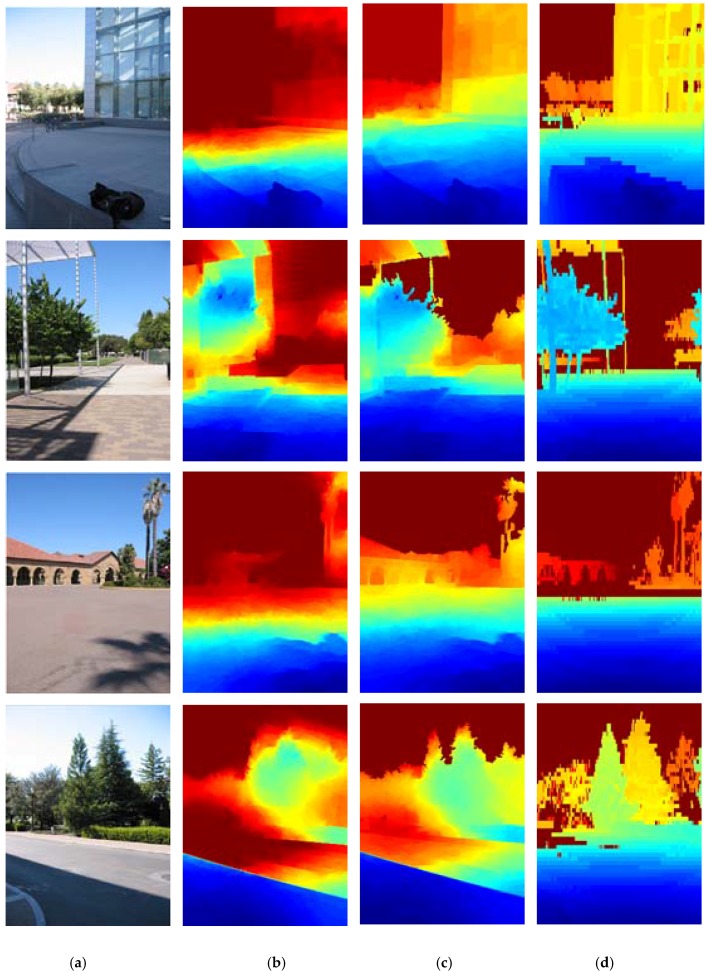
Qualitative comparison of depth reconstruction via the proposed approach and Unconstrained. Color indicates depth (red is far, blue is close). (**a**) Test images (**b**) Unconstrained (**c**) Proposed approach (**d**) Ground-truth.

In the experiments, depth maps are predicted via the CNN model with different constraint information. The results are shown in Table 1, where Unconstrained represents the model without integrating the semantic information and relative depth trends of local regions. Sematic_constrained represents the model with integrating only the semantic information. Local_constrained represents the model with integrating only the relative depth trends of local regions. Eucli_loss represents the model in which the loss function of the model replaces the CRF loss with a Euclidean loss and depth reconstruction becomes a regression problem as done in much existing work. A qualitative comparison of depth reconstruction with these methods is presented in Figure 3.

From the results illustrated in Table 1, the following considerations can be outlined.

(1) The method through Sematic_constrained can get more satisfactory results compared with Unconstrained, which demonstrates the semantic information is an effective cue for depth reconstruction.
(2) Likewise, the relative depth trends of local regions are helpful to depth reconstruction because the results via Local_constrained outperform Unconstrained.
(3) The errors of depth reconstruction through Eucli_loss are lower than Unconstrained. This is mainly because their loss functions are different. Eucli_loss uses a Euclidean loss as the loss function of the model. Unlike Eucli_loss, Unconstrained uses a pairwise CRF to establish the loss function, which can consider depth consistency and smoothness between the neighboring superpixels.
(4) As result of the semantic information, the relative depth trends and the pairwise CRF incorporated into the model, the proposed approach can get more satisfactory results than other methods.

### 3.2. The Experiments with Different Methods

To show the effectiveness of the proposed approach, several state-of-the-art methods are tested for comparison:

Saxena et al. [13]: The method learns the relation between image features and depth values using MRF. The image features including haze, texture variations and gradient, and shape- and location-based features are manually extracted and represented.

Liu et al. [14]: Based on Saxena et al. [13], Liu et al. [14] added semantic labels to guide depth reconstruction from a single image, but the method still depends on hand-crafted features.

Depth transfer [25]: The method is a non-parametric learning, which avoids explicitly defining a parametric model and requires fewer assumptions as in other methods [13,14]. Likewise, it still depends on hand-crafted features.

DC CRF [17]: In the method, depth prediction is formulated as a discrete-continuous optimization problem, which is solved via particle belief propagation in a graphical model.

DCNF [23]: The method performs depth reconstruction by jointing CNN and CRF. Unlike the proposed approach, the method does not consider semantic information and local detail information from images.

**Figure 4:**
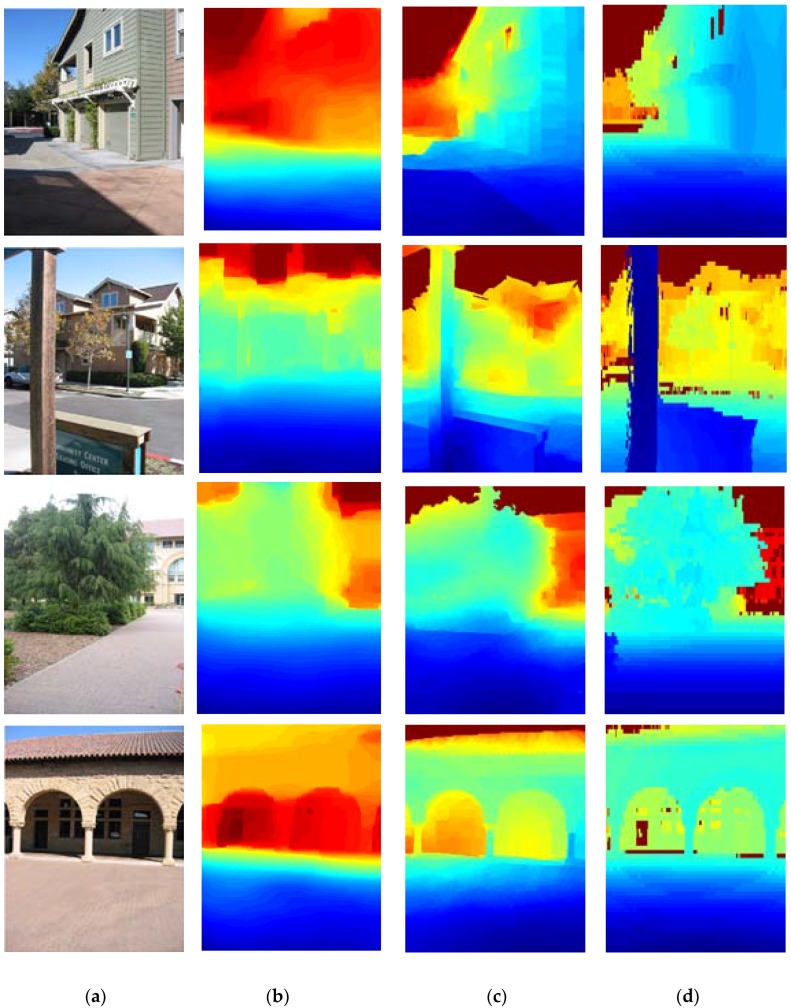
Qualitative comparison of depth reconstruction via the proposed approach and depth transfer [25]. (**a**) Test images (**b**) depth transfer [25] (**c**) Proposed approach (**d**) Ground-truth.

The results of these methods are shown in Table 2. A qualitative comparison of depth reconstruction is presented in Figure 4.

From the results illustrated in Table 2, the following considerations can be noted:

(1) DCNF [23] and the proposed method significantly outperform the other four methods. This is mainly because the other four methods predict depth maps from a single image via hand-crafted features. Instead, DCNF [23] and the proposed method use the CNN model which can automatically learn a high-level of feature representation without any manual intervention.
(2) The proposed approach can get more satisfactory results than DCNF [23], because the proposed approach integrated into the semantic information and relative depth trends of local regions.

**Figure 5:**
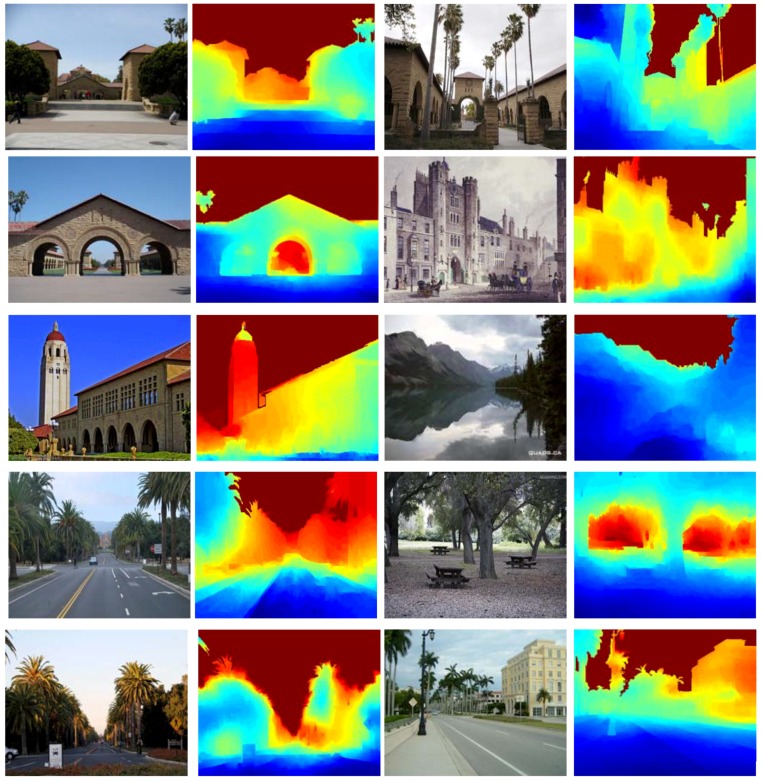
Depth reconstruction for images from the Internet.

Besides, depth maps are reconstructed for some images not in the Make3D dataset, but from the Internet, which further demonstrate the effectiveness of the proposed approach in Figure 5.

## 4. Discussion

Through the experiments, it is observed that the proposed method is successful at depth reconstruction from a single image with satisfactory accuracy. The proposed approach for depth reconstruction uses a unified CNN framework, joining the advantages of the CNN and the continuous pairwise CRF model. On the one hand, it can the automatically learn hierarchical feature representation of the image via CNN model rather than hand-crafted mode. On the other hand, depth reconstruction is formulated as a CRF learning problem rather than a regression problem due to the loss function that uses a continuous pairwise CRF instead of a Euclidean loss. In the continuous pairwise CRF, the depth consistency and smoothness of neighboring superpixels are considered. Additionally, the unified framework incorporates into the sematic information and relative depth trends of local regions, which can be helpful to resolve depth ambiguities and provide more local details in the image. Therefore, depth reconstruction through the proposed approach is effectiveness and has some improvements.

## 5. Conclusions

In this paper, the development and implementation of a new approach for depth reconstruction from a single image is presented. A unified framework joining a CNN and pairwise CRF model is used to obtain depth information. A particular feature of the approach is that semantic information and relative depth trends of local regions are integrated into the unified framework. A series of experiments on Make3D dataset are presented in this paper. The experiments with different constraint information demonstrate that the semantic information, the relative depth trends of local regions and CRF model are helpful to depth reconstruction from a single image. The experimental results show that the proposed method is effective and suitable for depth reconstruction.

## Figures and Tables

**Table 1:** Errors of depth reconstruction with different constraints.

Methods	C1 Error			C2 Error		
	Rel	Log_10_ (m)	Rmse (m)	Rel	Log_10_ (m)	Rmse (m)
Eucli_loss	0.366	0.137	8.63	0.363	0.148	14.41
Unconstrained	0.312	0.113	9.10	0.305	0.120	13.24
Semantic_constrained	0.291	0.109	8.74	0.287	0.114	12.10
Local_constrained	0.295	0.105	8.53	0.291	0.109	11.95
Proposed approach	0.260	0.092	7.16	0.245	0.103	10.07

**Table 2:** Quantitative comparisons with other methods.

Methods	C1 Error			C2 Error		
	Rel	Log_10_ (m)	Rmse (m)	Rel	Log_10_ (m)	Rmse (m)
Saxena et al. [13]	-	-	-	0.370	0.187	-
Liu et al. [14]	-	-	-	0.379	0.148	-
Depth transfer [25]	0.355	0.127	9.20	0.361	0.148	15.10
DC CRF [17]	0.335	0.137	9.49	0.338	0.134	12.60
DCNF [23]	0.312	0.113	9.10	0.305	0.120	13.24
Proposed approach	0.260	0.092	7.16	0.245	0.103	10.07

**Figure A1:**
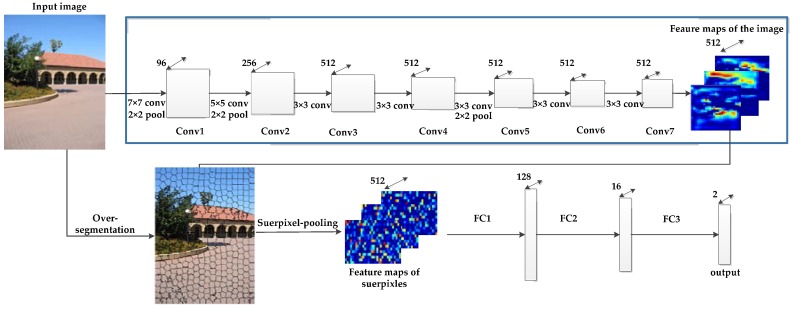
The CNN architecture in the unary part.

## Appendix A

The CNN architecture in the unitary part and some implementation details for the proposed approach are shown in this section.

### Appendix A.1. CNN Architecture in the Unary Part

The structure of the CNN model in the unary part is shown in Figure A1. The input image is first fed into seven convolutional layers (Conv1 … Conv7) to produce the convolutional feature maps of the image. Then these feature maps are fed to a superpixel pooling layer to transfer into the convolutional feature maps of the superpixels, which are followed by three fully-connected layers. The outputs of the model are the depth at the center of the superpixels and the normalized scale factors.

### Appendix A.2. Implementation Details of the Experiments

In the experiments, the superpixels in the images are firstly over-segmented by Simple Linear Iterative Clustering (SLIC) [26], which clusters pixels in the combined five-dimensional color and image plane space to efficiently generate compact, nearly uniform superpixels. For the Make3D dataset in this paper, the minimum size of the extracted superpixels is set as 10. To generate depth templates, the normalized depth maps of the superpixels in the same sematic label are clustered by Affinity Propagation (AP) [27]. During training and testing, the ground-truth depth values are transferred into log-space. The proposed unified network is trained via stochastic gradient decent with momentum of 0.9, and weight decay of 0.0005. The learning rate is initialized as 0.001, and divided by 2 after 10 cycles. Weights for the convolution layers Conv1 ... Conv5 are initialized by the pre-trained CNN-S model [28].The weights of the other layers are randomly initialized with standard deviation 0.01. In the actual scene, the photograph distance from the sky, that is much larger than the other objects, can be approximated as infinity. Thus, the depth of sky regions in the image can be directly assigned as a maximum value.
